# Multi-Device Parallel MRI Reconstruction: Efficient Partitioning for Undersampled 5D Cardiac CINE

**DOI:** 10.3390/s24041313

**Published:** 2024-02-18

**Authors:** Emilio López-Ales, Rosa-María Menchón-Lara, Federico Simmross-Wattenberg, Manuel Rodríguez-Cayetano, Marcos Martín-Fernández, Carlos Alberola-López

**Affiliations:** Laboratorio de Procesado de Imagen, Universidad de Valladolid, Campus Miguel Delibes sn., 47011 Valladolid, Spain; rosamaria.menchon@uva.es (R.-M.M.-L.); fedsim@uva.es (F.S.-W.); manrod@tel.uva.es (M.R.-C.); marcma@uva.es (M.M.-F.)

**Keywords:** cardiac CINE, parallel computing, multi-GPU, multi-device, MRI reconstruction, compressed sensing

## Abstract

Cardiac CINE, a form of dynamic cardiac MRI, is indispensable in the diagnosis and treatment of heart conditions, offering detailed visualization essential for the early detection of cardiac diseases. As the demand for higher-resolution images increases, so does the volume of data requiring processing, presenting significant computational challenges that can impede the efficiency of diagnostic imaging. Our research presents an approach that takes advantage of the computational power of multiple Graphics Processing Units (GPUs) to address these challenges. GPUs are devices capable of performing large volumes of computations in a short period, and have significantly improved the cardiac MRI reconstruction process, allowing images to be produced faster. The innovation of our work resides in utilizing a multi-device system capable of processing the substantial data volumes demanded by high-resolution, five-dimensional cardiac MRI. This system surpasses the memory capacity limitations of single GPUs by partitioning large datasets into smaller, manageable segments for parallel processing, thereby preserving image integrity and accelerating reconstruction times. Utilizing OpenCL technology, our system offers adaptability and cross-platform functionality, ensuring wider applicability. The proposed multi-device approach offers an advancement in medical imaging, accelerating the reconstruction process and facilitating faster and more effective cardiac health assessment.

## 1. Introduction

Dynamic cardiac magnetic resonance imaging (cMRI), and particularly, cardiac CINE, has become an indispensable tool in modern cardiovascular medicine [1]. Its preeminence is due to its ability to provide evidence-based assessments essential for the diagnosis and treatment of cardiovascular disorders. The accuracy and level of detail it offers enable medical professionals to comprehensively assess cardiac function and detect subtle abnormalities indicative of pathologies [2]. The reliability of cMRI as a diagnostic tool depends on meeting stringent criteria for spatiotemporal resolution and image quality.

In this context, our focus is on 5D cMRI [3,4], an advanced method for free-breathing (FB) acquired data that incorporates an additional temporal dimension for the respiratory phase, resulting in three spatial dimensions and two pseudo-temporal dimensions corresponding to the cardiac and respiratory phases. FB cMRI offers significant advantages. Firstly, it enhances patient comfort by eliminating the need for breath-holding, which is particularly crucial for patients with respiratory limitations or in critical conditions. Additionally, the efficiency of acquisition time is markedly improved, as there are no pauses for breath-holding, reducing the overall duration of the cMRI session and increasing patient tolerance.

However, these advantages are accompanied by an increase in the volume of data generated, presenting new challenges. On the other hand, in the context of the ongoing evolution of medical imaging paradigms, the quest for higher image resolution exponentially pushes this increase in data volume. Although this expansion enhances diagnostic capabilities, it introduces significant computational challenges. These challenges raise the time and computational demands for proper processing, making MRI reconstruction increasingly complex. Nevertheless, the diagnostic goals of reconstruction require rapid computational processing. This situation has driven the adoption of various strategies for accelerating both the acquisition and the reconstruction processes [1]. This emphasis turns attention towards the search for alternative methods to improve the speed of advanced iterative algorithms in parallel computing environments.

In this scenario, we should also note that the integration of artificial intelligence (AI) and deep learning methods has marked significant advances in MRI reconstruction [5,6], learning from large image datasets to enable faster reconstructions than traditional methods. Classical methods based on optimization, however, still maintain their interest since they can drive physics-informed learning-based solutions.

In this work, we propose a multi-device system capable of performing the entire 5D cardiac reconstruction process in multi-GPU configurations.Due to current memory limitations in a single GPU, implementing the entire reconstruction process on a single unit may not be feasible. Therefore, our alternative is oriented towards the use of a multi-device procedure while maintaining parallelization power. Despite this, implementation in a multi-device system is accompanied by several difficulties, particularly due to data dependencies, which arise because of the parallelization of the reconstruction algorithms.

In addressing the dependency issues associated with iterative algorithms, we have found a viable solution in the NESTA algorithm [7]. This first-order method is a fast and accurate algorithm that uses a nuanced averaging of iterations to improve the convergence of standard gradient descent algorithms. Our proposal is based on the hypothesis that it is possible to decompose the NESTA global optimization process—used in solving the minimization problem—into a series of smaller-scale local optimizations problems. This decomposition would not affect the quality of the resulting images and would be executed on subsets of data obtained by splitting the original data along some of their dimensions.

The implementation of this approach would allow for the effective parallelization of the algorithm. Moreover, by combining our method with the acceleration techniques already documented in the literature, we could further reduce the time required for reconstruction. This method not only improves efficiency in MRI reconstruction but also addresses the challenge of GPU memory limitations when dealing with large datasets.

To complement this strategy, we have opted for OpenCL technology [8] for the implementation of our system, thereby ensuring the portability of the code across a wide variety of platforms. Furthermore, we emphasize the importance of intelligent task allocation among the available devices in a multi-device system. Such distribution allows us to overcome the memory constraints of GPUs and further optimize reconstruction times. In this context, we highlight the adoption of the OpenCLIPER framework [9], which facilitates efficient resource management across the system’s computational devices. The flexibility of this framework allows us to utilize any computational device in the execution of parallel computations.

The remainder of this paper is structured as follows: Section 2 presents a concise summary of related research in MRI reconstruction using GPUs. Section 3 discusses the data employed and explains the methods used, along with details concerning the multi-device process and the computational operators used. Section 4 presents the results. Section 5 includes a brief discussion of these findings. Finally, Section 6 presents the main conclusions. An appendix with detailed explanations of the problem to be solved can also be found at the end of this article.

## 2. Background

Parallel computing environments commonly include Graphics Processing Units (GPUs) used for high-performance parallel computations. For example, Wang et al. [10] report that the integration of GPUs in MRI reconstruction has seen a notable increase as evidenced by the growth of related academic publications. This trend is attributed to the substantial improvements in MRI processing speed facilitated by the adoption of GPU technology. However, this speed increase is achieved by individually optimizing some reconstruction steps within the (single) GPU pipeline.

These optimizations, compiled into available and usable libraries, depend on the technology employed for their implementation. Among these are CUDA (a proprietary technology of NVIDIA widely used by the community) and OpenCL (a technology with an open-source API). Thus, many MRI reconstruction methods have utilized these libraries in their internal procedures to facilitate speedups. However, to take full advantage of the GPUs used in these calculations, it is necessary to redesign the algorithms to adapt them to the characteristics of GPUs.

Building upon these developments, the acceleration strategies facilitated by GPUs have been increasingly applied to Compressed Sensing (CS) MRI reconstruction, which is pivotal in addressing the heightened computational demands. Various studies have demonstrated the capability of GPU acceleration in conjunction with this technique, significantly improving the speed of MRI reconstruction [11,12,13].

It is essential to employ advanced tools and methods to address more efficiently and accurately the complexities presented by reconstruction algorithms. The use of highly optimized libraries is elemental to enhance the efficiency of this process. However, a notable challenge arises due to the inability of reconstruction algorithms to be fully accommodated in the memory of a single GPU. This is primarily because the growing volume of data approaches the limits of current GPU memory capabilities. This limitation, along with the algorithms used, results in parallel optimization that is often confined to individual operations. Therefore, overcoming this limitation represents a considerable challenge. Moreover, this bottleneck is especially pronounced in the realm of 5D cMRI reconstruction. Given this situation, it becomes essential to find a solution that efficiently leverages the computational power of multi-device architectures. Regarding this aspect, Table 1 shows a summary of research related to MRI reconstruction using the multi-GPU system.

Almost half of the multi-GPU systems presented in the table, such as [14,19,21], perform 2D reconstructions. These reconstructions are parallelized at the thread level within GPUs. Since they do not have temporal dimensions and, due to their dimensionality, their size is not large, they do not present problems that require synchronisms in the multi-GPU systems in which they are implemented. In the case of [17], the data used, despite being 2D, do have a temporal dimension, so in this case they need synchronization. However, they report an overload that leads them to relax the temporal regularization they use, so they reconstruct frames without completely reconstructing the previous ones on which they depend. On the other hand, all of them perform CUDA implementations, so they depend on NVIDIA technology and hardware to operate.

In the other half of the table, we can observe 3D reconstruction implementations. However, some of them, such as [16,18,20] do not need synchronization since they perform parallelization using decoupled 2D reconstruction. Moreover, these implementations correspond to static 3D data. However, the study of Lecoeur, B. et al. [15] presents and implements multi-GPU MRI 3D+t reconstruction (with a respiratory dimension). According to their methodology, they use a CPU and GPU parallelization system. The multi-GPU parallelization they report is due to the stack-of-stars nature of the data, where the data are independent between *z*-axis slices. This allows them, after running on CPUs part of their algorithm, to parallelize 2D+t sets on the different devices independently since they have no dependencies. This means that their system does not have synchronization between the devices used. In addition, they depend on CUDA, so they are also limited in this way.

While we have identified systems in the literature that parallelize 3D+t MRI [15], these systems do not demonstrate parallelization that spans multiple GPUs for full 5D cardiac data reconstruction (incorporating all three spatial dimensions in addition to cardiac and respiratory dimensions) with the same depth as our system proposes. Specifically, our system explicitly addresses workload distribution and inter-task synchronism for the efficient use of multiples GPUs, which represents a substantive contribution with respect to existing systems. While previous work focuses on the reconstruction of lower-dimensionality data without the need for complex synchronization between devices, our system handles the added complexity of 5D data and ensures consistent and coordinated reconstruction across multiples GPUs, regardless of their manufacturer. The key distinction of our approach lies in how load-splitting and synchronization mechanisms between GPUs are employed to effectively address this high dimensionality and complexity, an aspect that, to the best of our knowledge, has not been explicitly addressed by other solutions using multiple GPUs.

## 3. Materials and Methods

### 3.1. Data and Hardware Used

In our research, we employ synthetic data from the XCAT numerical phantom v2 [22]. A FB bSSFP acquisition was simulated, guided by the following parameters: TR/TE values at 3.0/1.5 ms, a flip angle set at 60°, field of view (FOV) of (168 mm)^3^, and a spatial resolution of 2 mm^3^. This simulation encompassed multi-coil data, with a total of 16 coils. For the study, the fully sampled data were undersampled retrospectively, utilizing the 3D variable density spiral-like Cartesian sampling scheme (VD-CASPR) [23] with different acceleration factors (AF): 4, 8 and 10. This method was chosen to obviate the need for gridding processes during subsequent reconstruction. The data were categorized into 20 distinct cardiac and 4 respiratory phases. This categorization led to a total of 80 reconstructed volumes.

To evaluate the performance of the results, we mainly used a dedicated hardware system (server 1) consisting of 4 Nvidia RTX A5000 GPUs [24] (each equipped with 24 GB of VRAM), 2 AMD EPYC 7513 CPUs [25] (32 cores/64 threads each) at 2.6 GHz and 1 TB of RAM. We also used a separate dedicated hardware system to complement the memory tests (server 2) consisting of 2 Nvidia GPUs (Quadro RTX 6000 24 GB VRAM [26] and Quadro RTX 5000 16 GB VRAM [27]), 2 Intel Xeon E5-2697 CPUs [28] (17 cores, 34 threads each) at 2.3 GHz and 500 GB RAM.

### 3.2. Method

In this work, our principal contribution is the integration of the entire reconstruction process within a multi-device system through the parallelization of the problem. In this way, we employ several devices at the same time, which provides substantial speed gains while preserving the quality of the reconstructed image. In this section, our focus is to detail the data-partitioning strategy, as well as the resource management and dependency handling in the 5D system we propose. The process is designed to be flexible, allowing it to be hosted on the device of choice within the system. This adaptability enables configuration for exclusive GPU usage or, if required, for utilizing both the GPU and the CPU as integral components of the system. To fully grasp this methodology, it is essential to understand the 5D CS reconstruction process. The minimization problem under consideration is expressed in Equation (1):

(1) $$\hat{m}=\underset{m}{argmin}\frac{1}{2}{∥Em-y∥}_{l_{2}}^{2}+\lambda {∥\Phi m∥}_{l_{1}}$$

where y denotes the acquired k-space data, m is the image to be reconstructed, E represents the encoding operator (incorporating coil sensitivity maps, Fourier Transform, and the sampling matrix), and Φ represents a sparsifying transform. The objective function in Equation (1) consists of a squared $l_{2}$-norm term, corresponding to the data fidelity term, and the typical $l_{1}$-norm constraint used in CS. Therefore, the regularization parameter λ establishes a trade-off between data consistency and the sparsity of the solution.

Our hypothesis posits that this global optimization process can be divided into smaller local optimization problems without compromising the quality of the reconstructed images. We have employed the iterative NESTA algorithm [7] to solve the optimization problem in Equation (1). NESTA uses the well-known Huber function as an approximation of the $l_{1}$-norm. For the sparsifying transform Φ, we have selected the temporal total variation (tTV). Therefore, the objective function in Equation (1) is evaluated at each iteration of the algorithm as follows:

(2) $$f\left(m^{k}\right)=\frac{1}{2}{∥Em^{k}-y∥}_{l_{2}}^{2}+\lambda \cdot f_{\mu }\left(\Phi m^{k}\right)$$

where *k*-index denotes the iteration number, and $f_{\mu }$ stands for the Huber function.

The gradient of the objective function is also computed iteratively in the following form:

(3) $$\nabla f\left(m^{k}\right)=E^{H}\left(Em^{k}-y\right)+\lambda \cdot \underset{\Lambda }{\underbrace{\Phi^{H}f_{\mu }^{\prime }\left(\Phi m^{k}\right)}}$$

where $f_{\mu }^{\prime }$ represents the gradient of $f_{\mu }$ (defined as indicated in Equation (A2)), and $\left\{E^{H},\Phi^{H}\right\}$ stand for the corresponding adjoint operators.

The critical aspect of problem division lies in the parallelization of the second term of the gradient calculation (Λ), i.e., the gradient of the regularization term. The parallelization of this term is complicated by temporal dependencies present in the cardiac and respiratory dimensions due to the use of tTV (see Appendix A). Addressing this issue involves two key aspects: data partitioning and dependency management.

It should be noted that the parallelization of NESTA is comprised of the modification of the sparsifying transform, inter-device communication, and the synchronizations necessary to maintain the integrity of the iterative nature of the algorithm. Moreover, the calculations of both the $l_{1}$-norm and $l_{2}$-norm are performed with the data held by the device on which the algorithm is running. The global optimization problem becomes smaller local optimization problems. Even so, the internal steps of the NESTA algorithm remain unchanged.

#### 3.2.1. Data Partitioning

As we have previously noted, large data volumes often encounter memory limitations in devices. Consequently, rather than processing the entire volumes, we choose to subdivide the data along temporal dimensions based on the specifications of the available devices (predominantly GPUs). Specifically, we prioritize GPUs with the highest amount of VRAM available in the desired configuration. Typically, these GPUs tend to be those with the highest computational power. We have decided to use this strategy in order to balance compute capacity and workload. In this way, a balanced computational load can be maintained between GPUs. This implies that systems can effectively utilize GPUs with a wide range of configurations, encompassing both specification differences and diverse manufacturers. However, when utilizing certain system configurations, it is important to consider that using devices with significantly lower computational power may dictate the time required to solve the reconstruction problem.

Data subdivision along temporal dimensions can be implemented in either the cardiac or the respiratory dimension. Given that both approaches are similar and that the cardiac dimension usually encompasses more phases than the respiratory one, we have chosen to perform the subdivision in the cardiac dimension. This approach allows us to minimize dependencies among the data subsets housed in the devices, as explained in the next section, and to maximize memory usage.

**Figure 1:**
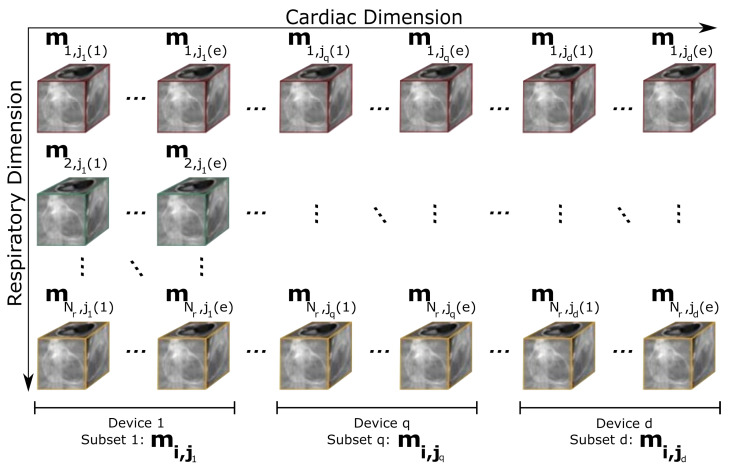
Schematic illustration of data partitioning along the cardiac temporal dimension, distributed across multiple devices to enable parallel reconstruction. In this schema, $m_{i,j_{q}}$ represents the subset of cardiac phases and their corresponding respiratory phases processed by device *q*. The colors (red, green and yellow) serve to visually distinguish between the different respiratory phases.

The subdivision of data along the cardiac temporal dimension is illustrated in Figure 1, where each $m_{i,j}$ represents a 3D volume with a specific state within the respiratory ($1\leq i\leq N_{r}$) and cardiac ($1\leq j\leq N_{c}$) dimensions, $N_{r}$ being the total number of respiratory phases and $N_{c}$ the total number of cardiac phases. Thus, the subset $m_{i,j_{q}}$ is allocated to a given device *q* (with 1≤q≤d and *d* is the total number of devices), where the subscript i denotes the whole set of respiratory phases, and $j_{q}$ stands for the subset of consecutive cardiac phases (from $j_{q}\left(1\right)$ to $j_{q}\left(e\right)$) in the device *q*. Note that, with this notation, $j_{1}\left(1\right)=1$ and $j_{d}\left(e\right)=N_{c}$. Figure 1 provides a simplified representation, which does not consider the temporal dependencies inherent to the problem.

#### 3.2.2. Data Dependencies

The necessity to address dependency issues arises from the fact that neglecting them during the parallelization of the second term in Equation (3) (Λ) would lead to inconsistencies in the reconstructed images, resulting in a degradation of the reconstruction quality.

**Figure 2:**
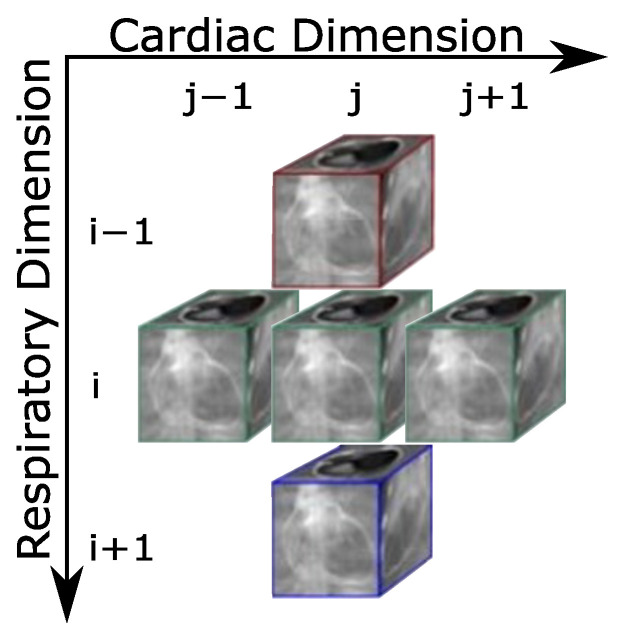
Dependencies of a frame with respect to neighboring frames in the gradient evaluation (term $\Lambda_{i,j}$ in Equation (A1)). As in the Figure 1, the colors (red, green and blue) serve to visually distinguish between the different respiratory phases.

As discussed in the previous section, dataset division is performed along the cardiac dimension to minimize the dependencies between the resultant subsets. These dependencies arise due to the application of the tTV operators (denoted as Φ and $\Phi^{H}$) in the gradient calculation (Λ in Equation (3)). The dependencies in question comprise the 3D data volumes in the cardiac and respiratory phases. Given that the calculation of Λ is defined as shown in Equation (A1), the dependencies for a specific volume are on the volumes of the immediately adjacent phases. A more elaborate explanation of these dependencies is provided in Appendix A. Figure 2 graphically depicts these dependencies for a given frame $m_{i,j}$ within any given subset (see Figure 1). As commented above, the indices *i* and *j*, with $1\leq i\leq N_{r}$ and $1\leq j\leq N_{c}$, correspond to the temporal dimensions of the respiratory and cardiac phases, respectively.

**Figure 3:**
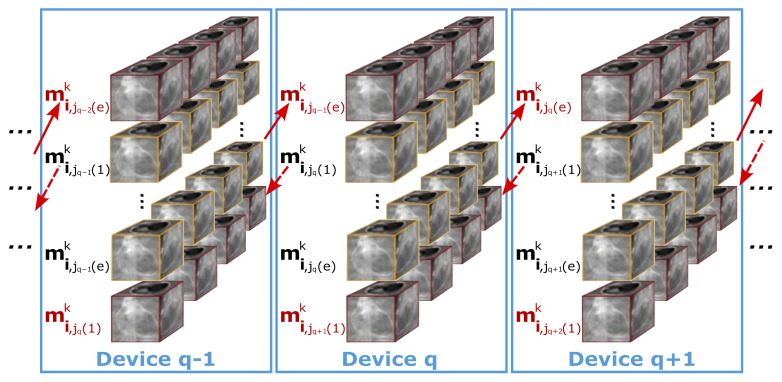
Schema of dependencies in the parallel multi-device reconstruction process. Each $m_{i,j_{q}}^{k}$ represents the subset of all respiratory phases for a single cardiac phase at the *k*-th iteration in the device *q*. The red color indicates the dependencies between each device and the others, illustrating the interconnection necessary for parallel processing. The yellow color, on the other hand, delimits the data processed by the device itself, highlighting segments of the device-specific data set.

Parallelization through data division results in 3D volumes at the boundaries of such division having their dependencies spread across different devices. Figure 3 illustrates the structure of these dependencies in the parallel multi-device CINE reconstruction process. At a synchronized *k*-th iteration, device *q* processes the subset $m_{i,j_{q}}^{k}$ of volume images. Therefore, each device holds a portion of the overall dataset along with its dependencies. As illustrated in Figure 3, device *q* contains the subset from $m_{i,j_{q}\left(1\right)}^{k}$ to $m_{i,j_{q}\left(e\right)}^{k}$, as well as the 3D volumes corresponding to the previous cardiac phase ($m_{i,j_{q-1}\left(e\right)}^{k}$, with $j_{q}\left(1\right)=j_{q-1}\left(e\right)+1$) from device q−1, and to the next cardiac phase ($m_{i,j_{q+1}\left(1\right)}^{k}$, with $j_{q+1}\left(1\right)=j_{q}\left(e\right)+1$) from device q+1. Data dependencies are indicated by arrows. This pattern is replicated across all intermediate devices (2≤q≤d−1). The first device manages its respective groups of volumes and additionally, the initial cardiac phase of the second device ($m_{i,j_{2}\left(1\right)}^{k}$). Conversely, the final device encompasses its assigned volumes as well as the last cardiac phase from the d−1 device ($m_{i,j_{d-1}\left(e\right)}^{k}$).

#### 3.2.3. Managing Data Dependency

To address the temporal data dependencies between devices, we have established runtime communication channels between GPUs using OpenCLIPER. This communication occurs indirectly among devices. As previously explained, each device maintains in its memory the dependencies of others. Except when using the CPU as a device, this memory is typically inaccessible to other GPUs. Therefore, it is necessary for each GPU to provide the data that other devices depend on so that all processes are correctly executed. There are two main reasons for maintaining indirect communication between devices: firstly, to the best of our knowledge, direct communication between GPUs is currently difficult to achieve among the wide variety of devices available in the market. Secondly, the devices that allow it usually require proprietary technology, such as NVLink [29]. As previously mentioned, one of our research objectives is to maintain code portability across a broad range of platforms; this means that our solution should enable device communication in configurations with multiple devices from various manufacturers.

**Figure 4:**
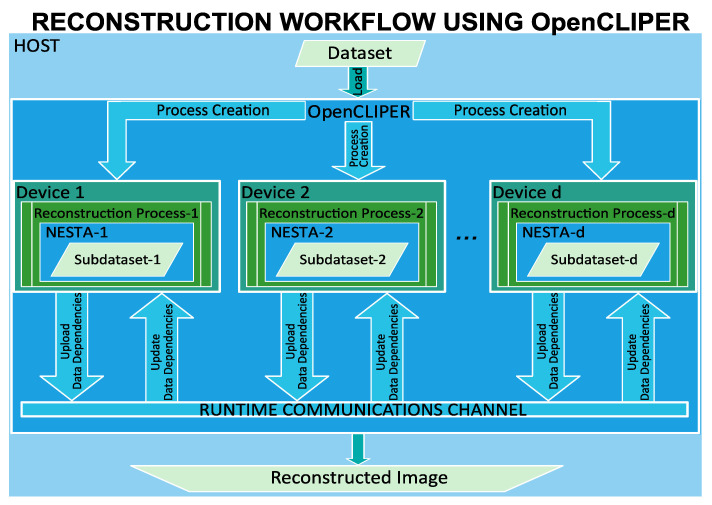
Schematic representation of the reconstruction workflow using OpenCLIPER. The process initiates with data loading into OpenCLIPER on the host, followed by the creation and distribution of reconstruction processes and data subsets to individual devices. Each device utilizes a modified NESTA algorithm for reconstruction.

Figure 4 illustrates the entire reconstruction workflow using OpenCLIPER, showing that the 5D CINE reconstruction operates as a set of internal processes within the devices. OpenCLIPER, which is the central coordinating unit that oversees the execution, initiates these processes by allocating the data subsets and specifying the interdependencies to be communicated among the devices involved. Once initialized, each device functions autonomously. During the reconstruction, the devices interact through a runtime communication channel—a network of buffers allocated for each dependency—ensuring synchronized data exchange. Within this framework, devices upload the dependencies required by others into designated buffers, and subsequently retrieve any dependencies they need from these shared resources. OpenCLIPER manages this buffer network, coordinating the distribution and collection of dependencies. After dependencies are addressed, the processes on each device proceed independently until completion. When all devices have finished their tasks, the composite image is fully assembled and available in the system.

The success of the algorithm’s execution relies on the system of indirect communication that manages temporal dependencies. This component is essential for preserving the integrity of the process’s iterative nature. Proper synchronization (background material about thread programming can be found in [30]), facilitated by mutexes and condition variables, ensures that dependencies are safely accessed and updated in real time, minimizing bottlenecks and aligning the start of each algorithmic step. This approach guarantees that all devices utilize the correct dependencies, allowing for synchronized progression and accurate gradient computation essential for the Λ calculation (Equation (A1)).

Before delving into the details of synchronization mechanisms, it is essential to understand that the NESTA algorithm is structured into two loops: an outer loop that controls the stages of the algorithm, and an inner loop that manages the iterations within each stage. Each stage (4 in our case) is designed to refine the $l_{1}$-norm approximation using different values of μ. These stages, allowing up to 100 iterations each, are tailored to ensure convergence.

**Figure 5:**
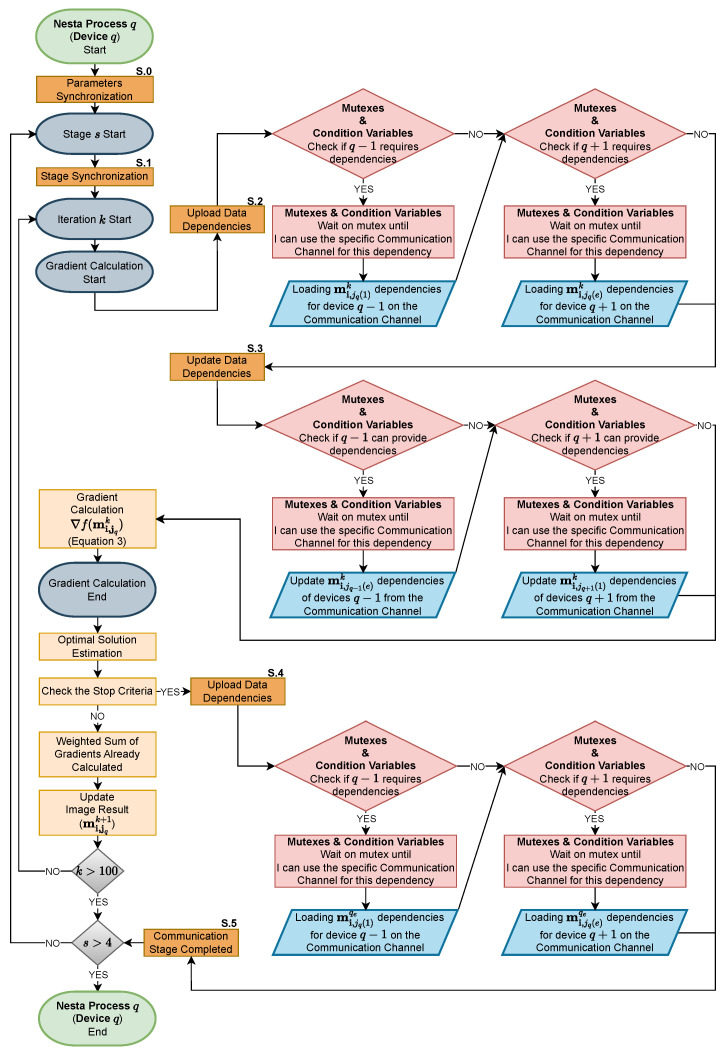
Flowchart depicting the reconstruction process on device *q*, where 2≤q≤d−1. This chart illustrates the management of data dependencies, the points at which synchronization occurs, and the use of mutexes and condition variables to ensure proper coordination between concurrent tasks. The notation $m_{i,j_{q}}^{e_{q}}$ with the superscript $e_{q}$ signifies the subset corresponding to the device’s final convergent iteration. Devices 1 and *d* behave similarly, but dependencies are not considered bilateral at this stage.

Turning to the specifics of synchronization, Figure 5 illustrates how, prior to entering these loops, devices share computed constants to initiate stages with values consistent with those in the global optimization version (see S.0 in Figure 5). To perform this synchronization, there is a mutex to manage the correct passage of constants values. Once the stage loop starts, stage-level synchronization is necessary to manage scenarios where some devices may require more iterations than others to achieve convergence (see S.1 in Figure 5). This synchronization ensures that devices do not share dependencies across different stages. There is a mutex that allows all devices to start the stages at the same time. Within the same stage, just before the gradient computation, devices share their data through respective communication channels tailored for each dependency (see S.2 in Figure 5). It should be noted that each dependency is associated with its own communication channel to avoid bottlenecks. In the same way, each communication channel, being shared by two devices, has its own mutex for correct access. Therefore, the use of mutexes specifically for each communication channel allows maximizing the amount of dependency transfers between different devices, avoiding unnecessary waiting. Subsequently, devices update their data by retrieving necessary dependencies from their specific communication channels (see S.3 in Figure 5). In this case, we are at the other extreme of independent communication channels, managed by the same channel-specific mutexes as the previous synchronization (S.2), whose implementation ensures correct access to dependencies. As previously mentioned, this process takes into account completed phases, allowing devices to rely on existing dependencies in memory when no new updates are available. When a device completes its tasks ahead of others, it updates its data for the last time in the stage *s* (see S.4 in Figure 5).The mutexes used in this synchronization are the same as those used for the S.2 and S.3 synchronizations. The superscript $e_{q}$ in place of *k* in $m_{i,j_{q}}^{e_{q}}$ indicates that this set corresponds to the final iteration (*e*) at which the device (*q*) has converged. Subsequently, the device informs the dependent devices that they should proceed with the latest updated data (see S.5 in Figure 5) to finalize their convergence. This synchronization has a general mutex that ensures communication that the device has finished its last iteration. Upon completing this mechanism, devices that have finished their stage wait to start the next stage with the rest of the devices. This ensures that all devices begin each stage simultaneously, relying on an accurate set of dependent data for each iteration.

## 4. Results

The results presented in this section are from a series of experiments conducted with the same dataset (see Section 3), using different acceleration factors: AF4, AF8, and AF10. These experiments were performed on a multi-device system configured in different ways: with CPU only, 2 GPUs, 3 GPUs, and 4 GPUs, resulting in a total of 12 distinct experiments. Each experiment was replicated 50 times to obtain the metrics discussed below.

As mentioned before, the reconstruction algorithm used consists of four internal stages of NESTA, with different values of parameter μ in the $f_{\mu }$ function for $l_{1}$-norm approximation, with each stage performing up to a maximum of 100 iterations to achieve convergence. The algorithm employs a stopping criterion based on the relative convergence of the objective function ($f(m^{k})$ in Equation (2)), activated after a minimum number of iterations (τ=7). It is necessary to mention that each device (*q*) calculates a $f(m_{i,j_{q}}^{k})$ value with the data it works with. This is evaluated at every iteration using the progress quotient:

(4) $$q_{p}^{k}=\frac{\bar{f}-f\left(m_{i,j_{q}}^{k}\right)}{\bar{f}}$$

where $\bar{f}$ is the average of the objective function in the last τ iterations of the algorithm. The algorithm halts when $q_{p}$ is less than or equal to a user-defined tolerance threshold, ensuring termination when the relative improvement of the objective function between iterations is minimal.

On the other hand, it is important to note that the CPU-only test was conducted through the system configuration set to use only the CPU using OpenCLIPER, employing the same parallelization techniques as those applied to the GPUs. However, unlike in the multi-device configurations, the CPU-only tests did not involve data division and temporal dependency management, and optimizations were conducted on a global scale rather than the local optimizations characteristic of multi-device approach. This approach ensures a more accurate comparison of the benefits offered by our proposal.

It is worth mentioning that the data volume prevents reconstruction using a single GPU due to memory constraints. To the best of our knowledge, and we believe this to be the primary reason, no other 5D reconstruction methods implemented on GPUs have been identified. This means that a direct comparison of our results with other 5D reconstruction tools is not feasible.

### 4.1. Reconstruction Times

**Figure 6:**
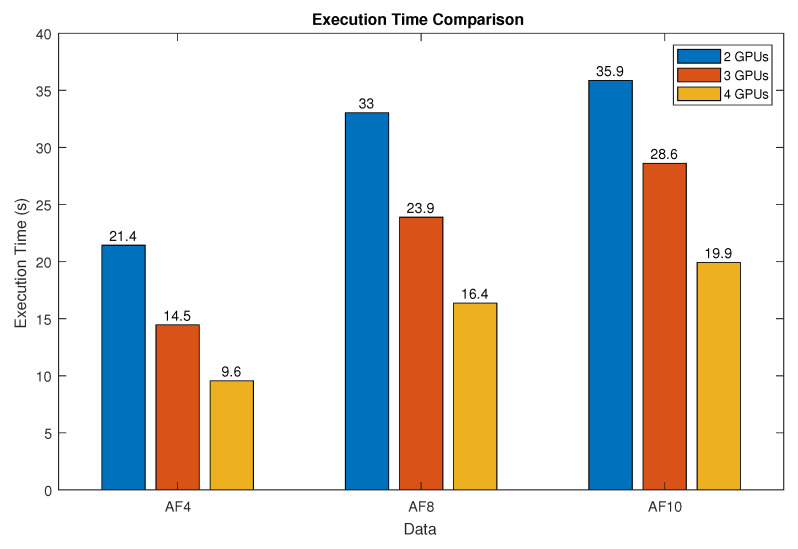
Comparison of reconstruction times for algorithms in different GPU configurations, showing the reduction in time as the number of GPUs used increases in the AF4, AF8 and AF10 datasets.

Table 2 and Figure 6 show the mean reconstruction times obtained for all the experiments. Intuitively, one would expect that employing multiple GPUs would reduce the reconstruction time proportionally depending on the number of devices used. The data confirm a substantial reduction in time when utilizing multiple GPUs as opposed to a single CPU. However, this reduction is not strictly proportional to the number of devices used for data with AF of 8 and 10. It is important to note that higher AFs require more time to converge because of the extensive subsampling involved. The increased number of iterations needed for convergence leads to a greater need for communication between devices for dependency management. Conversely, with a lower AF, the time reduction does seem to be directly proportional to the number of GPUs employed, attributed to the reduced number of iterations and, hence, a lesser need for communication among devices.

### 4.2. Global versus Local Optimizations

**Figure 7:**
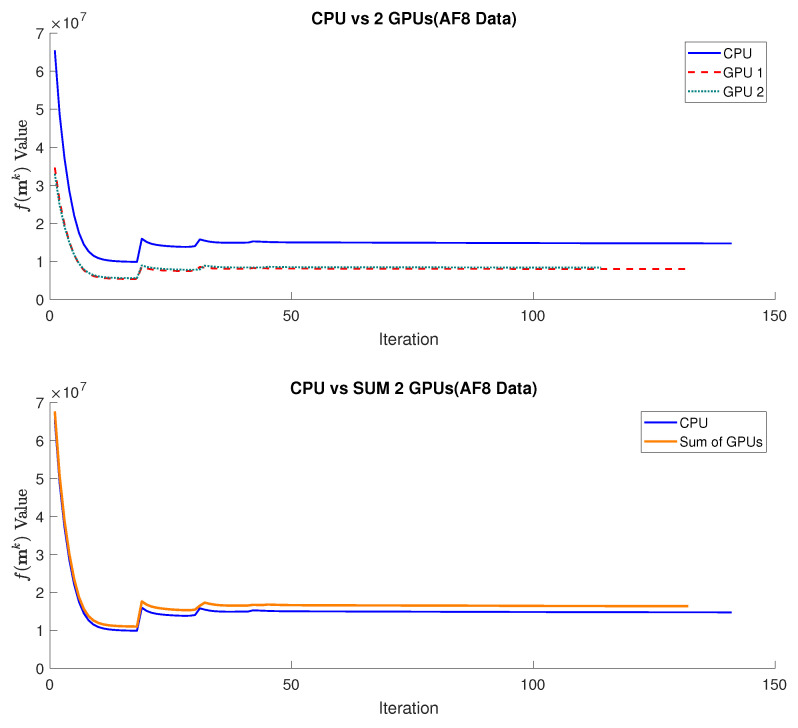
Comparison of the $f(m^{k})$ values obtained in the reconstruction using NESTA over the iterations required for convergence between the global optimization approach on a CPU and the parallel local optimizations approach on multi-device configuration using 2 GPUs and AF4 data.

The evolution of the objective functions value $f(m^{k})$ during the optimization process is depicted in Figure 7, contrasting the global optimization approach on a single CPU with our proposed method of parallel local optimizations on a multi-device configuration consisting of two GPUs. In the multi-device setup, each GPU computes its objective function $f(m_{i,j_{q}}^{k})$ and performs the gradient calculation as per Equation (3), utilizing its data subset and corresponding dependencies. A notable decrease in the value of $f(m_{i,j_{q}}^{k})$ is observed for the multi-device configuration when compared to the CPU. Furthermore, the aggregated values from the GPUs closely match the CPU curve, indicating that the results of the local optimizations on the GPUs are very similar to the global optimization performed by the CPU.

On the other hand, it can also be observed, together with Table 2, that the reconstruction requires fewer iterations to converge. However, we also observe an increase in the number of iterations required as the AF increases, which is expected due to the greater computational complexity introduced by a higher data subsampling rate in the reconstruction problem. Comparing the number of iterations (Table 2) required for the CPU execution against those required for GPU executions, we notice that they remain constant with minimal differences, except for the last step, where it can be seen that the multi-device version needs fewer iterations to converge than the CPU version. This suggests that our approach of parallel local optimizations maintains a similar performance to that observed in the global optimization approach.

### 4.3. Reconstruction Quality

**Figure 8:**
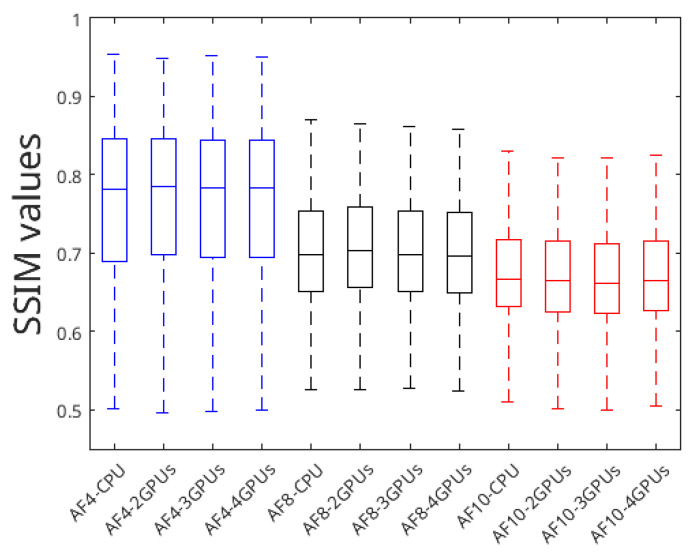
Box plot representation of SSIM distribution variability between CPU (global optimization) and multi-GPU (local optimization) reconstructions.

The next essential step is to validate whether the quality of the results obtained with the multi-GPU approach is comparable to the quality derived from CPU reconstruction. It is imperative to note that in our methodology, the minimization process is conducted through local optimizations, as opposed to the global optimizations inherent in CPU reconstruction. Given this difference, it is essential to evaluate the quality of the results. For this purpose, we consider the SSIM [31] as a relevant metric. Figure 8 presents a series of box plots that illustrate the distribution of SSIM values for different configurations with fully sampled image as reference. It is observed that the values obtained through local optimizations (multi-GPU configurations) are in line with those generated by the sequential global optimization process (CPU configuration).

Furthermore, in order to evaluate the robustness of the proposed multi-device reconstruction against noise, different levels of Gaussian noise were added to the synthetic MRI data (AF = 4), with SNR 24, 18 and 6 dB. Table 3 shows SSIM and PSNR values of the obtained reconstructions for the different configurations (two, three, and four GPUs) using the CPU reconstructions as reference. Both metrics indicate a high degree of similarity to the reference reconstruction.

### 4.4. GPU Usage Analysis

**Figure 9:**
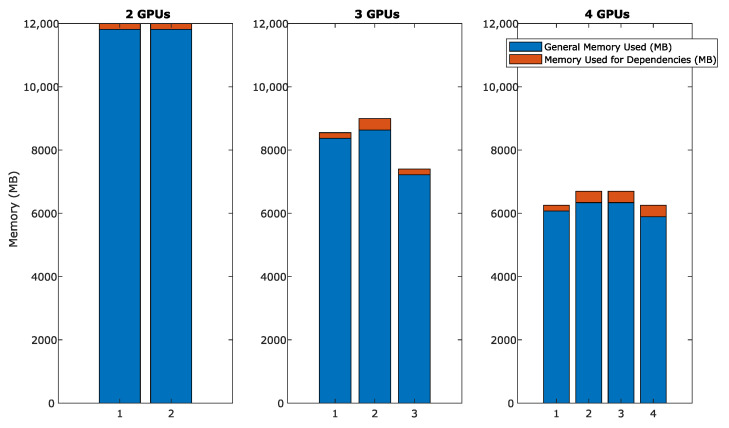
VRAM memory usage in configurations with 2, 3 and 4 GPUs (AF4 data) on server 1. Each bar represents the total memory used by a single GPU, subdivided into general memory used and additional memory used for dependencies.

Another important aspect to consider is the memory usage in different system configurations. To show these results, we have used server 1, where there are four GPUs with the same specifications. Table 4 provides a summary of VRAM memory usage and the number of frames processed in various GPU configurations applied to AF4 data. Given the particular arrangement of the data in the system, the amount of memory used to accommodate data of different acceleration factors does not vary, so the memory usage for the AF8 and AF10 sets is the same. The data overhead required for different configurations remains stable depending on the scenario in which the problem is to be divided. As discussed earlier, the GPUs processing the peripheral data sets interact only with one GPU due to their dependencies. In contrast, the GPUs assigned to the intermediate data sets (in configurations of three or more GPUs) will interact with two different GPUs. By requiring twice as many dependencies, twice as much overhead is caused. Even so, the overhead caused by the dependencies is proportionally low. In the case of the two-GPU configuration, the memory usage is identical since the specifications of the GPUs used are identical. In contrast, for the three-GPU configuration, we observe the scenario where the data partitioning is not exact. Hence, the first two GPUs have a higher load compared to the third one. Finally, with four GPUs, we can see how the distribution is more stable, where the GPUs with additional overhead due to dependencies with two different GPUs stand out. Figure 9 shows this behavior graphically. The blue color represents the amount of VRAM used in general, and red highlights the part needed for dependencies.

Another common scenario is that the devices used in the system configuration have different specifications. Table 5 shows how the data-partitioning algorithm has prioritized the most powerful GPU with the largest VRAM capacity in the system configuration to accommodate the largest volume of data. To show the behavior of this scenario, we have used server 2 where only 2 GPUs are available.

Finally, the utilization of GPUs observed in Table 6 shows the high performance and adaptability of our system across different configurations. In the two-GPU setup, we noted a peak utilization of 92% for both devices, with median values standing at 87% and 86%, respectively. Transitioning to the three-GPU setup, we recorded a maximum utilization of 90%, 89%, and 86%, with median values of 86%, 89%, and 77%, respectively. With the four-GPU setup, the maximum utilization values observed were 90%, 85%, 84%, and 87%, with median values of 84%, 82%, 80%, and 79%, respectively. The slight variation in median values suggest a workload balance. These data confirm a balanced distribution of computational load. The nuanced difference in utilization rates among the GPUs could be attributed to the inherent complexity of distributing tasks that vary in computation intensity.

## 5. Discussion

One of the primary points of discussion arises from the size of the datasets we have worked with. The considerable volume of these datasets poses a challenge for reconstruction on a single GPU, mainly due to memory limitations. This presents a dilemma, especially when juxtaposed with the growing demand for rapid, efficient, and high-quality reconstruction in the field of medical imaging. Our results suggest that distributing the computational load across multiple GPUs not only circumvents these hardware limitations but also achieves this without sacrificing the quality of the reconstruction. According to our results, the quality of the images is virtually indistinguishable from that of their traditional sequential counterparts. This reinforces the idea that multi-GPU configurations are a compelling solution to the challenges inherent in handling large MRI datasets.

It is also important to address the broader implications of our findings. As the field of medical imaging advances, there is an increasing demand for rapid and accurate reconstructions. In situations where clinical decisions depend on timely and precise imaging results, the time savings offered by conducting the entire reconstruction process within multi-GPU configurations using local optimizations could translate into tangible clinical benefits. Furthermore, the ability to deliver these results with minimal quality degradation represents a significant advance in the trajectory of MRI image reconstruction methodologies. Many of the emerging studies, as can be seen in Table 1, present proposals that address the problem. However, these approaches provide solutions to scenarios with low dimensionality (or none in terms of dynamic dimensions as far as cMRI is concerned). In addition, the parallelization presented by these proposals has a clear limit, as they focus on dividing those parts of the process that are independent of each other. Thus, they avoid addressing the problem of dependencies that arise when attempting to go further in the parallelization of reconstruction algorithms. In this way, our work presents a solution.

One of the strengths of the proposed system is that its design is geared towards interoperability with existing medical imaging workflows and industry standards. Our intention is for our tool to be versatile in its ability to be inserted into existing medical imaging ecosystems. We understand the importance of compatibility in today’s clinical environment, so the system has been designed to facilitate integration with prevalent medical imaging protocols. Through the implementation of a modular framework in OpenCLIPER, our system allows for easy incorporation of custom loaders and data savers. This means that users can extend the system to handle additional formats, such as HDF5 [32], DICOM [33], ISMRMrd [34], or any other emerging data format, thus ensuring long-term adaptability and relevance.

Regarding the potential limitations of this proposal, the NESTA algorithm provides good quality reconstructions at the cost of a much higher computational load than, for example, methods based on least squares. This prevents us from achieving a real-time reconstruction (such as [17], for instance), but when compared to typical acquisition times of about 15–20 min, the figures reported in Table 2 should not impede clinical practice when using our method. On the other hand, our proposal needs a powerful system too (a multi-GPU system), which may not be affordable for small clinics, but its typical cost is nevertheless about 100-fold less than the cost of an MRI scanner.

Another potential limitation of our proposal is that in order to perform inter-GPU communication, it is necessary to maintain buffers to the size of the dependencies in both host and devices. On the other hand, despite the high GPU utilization rates observed in the results, the data reveal potential areas for further optimization. The fact that not all GPUs are operating at or near maximum capacity at all times suggests that improvements in the allocation algorithms or the parallel processing architecture could lead to even more efficient use of resources. Optimizing these aspects could reduce processing times, enhancing the system’s responsiveness to real-time reconstruction demands.

Scalability must also be taken into account. As Amdahl’s law predicts, the serial load (including communication load) will inevitably exceed the computation load at a certain number of processing devices. From this point on, adding more GPUs will yield worse, rather than better, gains for the whole system.

Considering the economic and operational implications, we understand that the adoption of multi-GPU systems for medical image reconstruction presents a number of significant economic and operational implications for hospital environments. From an economic perspective, although the initial investment in a multi-GPU system is considerable, especially compared to less powerful hardware systems, it must be evaluated in the context of the total cost of an MRI scanner. Operationally, the implementation of multi-GPU technologies may require specific training for technical staff. However, with the proper interface and procedures, this transition can be facilitated.

In addition, the ability of our system to process a larger volume of data in times that do not impede clinical practice highlights its practical feasibility. This means that even if real-time reconstruction is not achieved, the improved efficiency in image reconstruction can facilitate greater patient throughput and more effective utilization of hospital resources, thus offsetting the initial investment through operational and clinical improvements.

In summary, the implementation of multi-GPU systems for medical image reconstruction promises substantial benefits in terms of image quality, operational efficiency and adaptability to different data types and reconstruction algorithms.

Another aspect we want to talk about is the environmental impact of using multi-GPU systems for MRI reconstruction, especially in terms of consumption and carbon footprint. In our research, the server used for testing consumes approximately 1000 watts during the reconstruction processes. This figure, while significant, must be contextualized within the larger picture of power consumption in medical imaging. Comparatively, an MRI scanner can consume 20 kWh ± 5 during operation [35], depending on the manufacturer’s specifications and the type of examination performed. This comparison highlights that although multi-GPU systems have a non-trivial energy impact, this is substantially lower compared to the consumption of MRI equipment.

To mitigate the environmental impact of our system, we propose several strategies:

- Algorithm Optimization: improve the efficiency of reconstruction algorithms to reduce processing time and thus energy consumption;
- Intelligent Energy Management: implement software solutions that enable more efficient energy management by GPU systems, adjusting the processing power to the real needs of the computation;
- Renewable Energy Used: encourage the use of renewable energy to power the data centers and servers that house these compute-intensive systems;
- Efficient Cooling: employ more energy-efficient cooling systems to decrease additional energy consumption.

All possible actions aimed at optimizing this consumption and reducing the carbon footprint are important steps towards sustainability. In addition, these strategies can offer long-term economic benefits by reducing operating costs.

## 6. Conclusions

This paper presents a comprehensive approach for parallelized 5D cMRI reconstruction using multi-device systems, overcoming the memory capacity limitations of individual GPUs and distributing load and synchronization between devices. Inter-device dependencies caused by data management are addressed through a runtime communication channel. Implementation using OpenCL ensures portability to a wide variety of platforms. The results obtained demonstrate a significant reduction in reconstruction time and maintenance of image quality as evidenced by improvements in processing times up to ×44.3 and results in SSIM and PSNR.

Although our proposal is focused on 5D cardiac CINE, it could also be applied to the reconstruction of other MRI modalities. In this case, parallelization process and inter-device communications have been optimized for the particular case of CS reconstruction with regularization based on temporal total variation, i.e., with temporal dependencies. Any problem that fits into this reconstruction scheme could be addressed by the proposed system. Furthermore, the system presents a versatile and potentially transformative platform for a wide range of applications in medical imaging and beyond. Its ability to handle high-dimensional reconstructions opens the door to its implementation in other areas of medical imaging that face similar challenges. For example, image reconstruction in neurology, oncology and interventional radiology, where data volume and the need for temporal and spatial resolution are critical, could benefit significantly from our methodology. In addition, the system architecture is based on flexibility and is not tied exclusively to proprietary technologies, so the system can operate on a wider range of hardware platforms, thus reducing costs and increasing the flexibility of advanced reconstruction technology. On the other hand, the system can be easily adapted to work with any other type of medical imaging. By changing the input data and the specific reconstruction algorithm, our system can be reconfigured to address a variety of medical imaging problems, thus offering a flexible and powerful solution to today’s medical imaging challenges. This adaptability underlines the relevance and potential of the system as a valuable tool in other medical specialties and research fields.

On the horizon of our research, we anticipate that technological advancement will bring lower-cost hardware to match the performance of current high-end solutions, thus enabling a significant reduction in the overall cost of processing systems.

Currently, work continues on the system, with a particular interest in exploring sampling patterns, especially radial sampling. In this way, we will be able to evaluate the adaptability of our parallel solution to various methodologies. In the future, we will study improvements of the synchronization mechanism and advanced load balancing strategies to further optimize performance on different hardware configurations. In addition, as a future line of research, we will investigate the use of compression techniques to increase memory efficiency and explore direct communication approaches between GPUs from different manufacturers. And, in relation to GPU utilization results, the identification of room for optimization underscores the ongoing need to refine our approach. We aim to unlock the full potential of multi-device reconstruction.

Another open front in our research is the integration of AI and deep learning for MRI reconstruction. Although our current focus is on parallelizing high-dimensional reconstruction, the incorporation of deep learning models could offer an avenue to overcome some of the inherent limitations of traditional methods, especially in terms of reconstruction speed and the handling of motion and noise artifacts.

On the software side, our work is focused on strengthening interoperability between OpenCL and CUDA, as well as pursuing initiatives for distributed processing, such as the development of PoCL 5.0, which bodes well for a more integrated and far-reaching research methodology.

## Figures and Tables

**Table 1:** Comparative summary of reconstruction research on multi-device systems.

Author [Ref.]	Year	Data ^a^	CPU	Parallelizated Dimension ^b^	Synchronization
Shafique, M. et al. [14]	2023	2D	Yes	Coil	No
Lecoeur, B. et al. [15]	2023	3D+t	Yes	Slice	No
Cuomo, S. et al. [16]	2018	3D	Yes	Slice	No
Schaetz, S. et al. [17]	2017	2D+t	No	Temporal	Yes
Piccialli, F. et al. [18]	2013	3D	No	Slice	No
Schaetz, S. et al. [19]	2012	2D	No	Coil	No
Murphy, M. et al. [20]	2012	3D	Yes	Slice	No
Zhuo, Y. et al. [21]	2010	2D	No	Slice	No

^a^ Parallel reconstruction on 2D or 3D MRI data. +t indicates dynamic data, i.e., with temporal dimension. ^b^ Dimension by which the problem is parallelized in the multi-GPU system.

**Table 2:** Reconstruction times of the 5D cMRI dataset (3D spatial with cardiac and respiratory dimensions) for different acceleration factors (AF).

AF	Devices Used	End-to-End Time ^a^	Recon Time ^b^	# Iterations ^c^
AF4	CPU	860.04 s	834.405 s	CPU: 15 + 10 + 9 + 61 = 95
AF4	Multi-GPU (2)	29.74 s (×28.9)	21.39 s	GPU 1: 15 + 10 + 9 + 54 = 88 GPU 2: 15 + 10 + 10 + 16 = 51
AF4	Multi-GPU (3)	21.79 s (×39.5)	13.65 s	GPU 1: 15 + 10 + 9 + 48 = 82 GPU 2: 15 + 9 + 9 + 16 = 49 GPU 3: 15 + 10 + 9 + 51 = 85
AF4	Multi-GPU (4)	19.42 s (×44.3)	9.56 s	GPU 1: 15 + 10 + 10 + 16 = 51 GPU 2: 15 + 9 + 9 + 16 = 49 GPU 3: 14 + 9 + 9 + 16 = 48 GPU 4: 15 + 10 + 10 + 33 = 68
AF8	CPU	1296.03 s	1270.33 s	CPU: 18 + 12 + 11 + 100 = 141
AF8	Multi-GPU (2)	42.62 s (×30.4)	33.04 s	GPU 1: 18 + 12 + 11 + 91 = 132 GPU 2: 18 + 13 + 13 + 70 = 114
AF8	Multi-GPU (3)	33.81 s (×38.3)	23.89 s	GPU 1: 18 + 12 + 11 + 84 = 125 GPU 2: 18 + 12 + 11 + 71 = 112 GPU 3: 18 + 13 + 17 + 54 = 102
AF8	Multi-GPU (4)	26.73 s (×48.5)	16.37 s	GPU 1: 18 + 12 + 11 + 76 = 117 GPU 2: 17 + 11 + 11 + 67 = 106 GPU 3: 17 + 11 + 11 + 59 = 98 GPU 4: 18 + 13 + 15 + 50 = 96
AF10	CPU	1344.21 s	1330.45 s	CPU: 19 + 12 + 11 + 100 = 142
AF10	Multi-GPU (2)	45.50 s (×29.56)	35.87 s	GPU 1: 19 + 12 + 11 + 100 = 142 GPU 2: 19 + 13 + 13 + 100 = 145
AF10	Multi-GPU (3)	37.01 s (×36.3)	28.61 s	GPU 1: 19 + 12 + 11 + 100 = 142 GPU 2: 19 + 12 + 11 + 93 = 135 GPU 3: 19 + 13 + 31 + 85 = 148
AF10	Multi-GPU (4)	31.18 s (×43.1)	20.83 s	GPU 1: 19 + 12 + 11 + 95 = 137 GPU 2: 18 + 12 + 11 + 89 = 130 GPU 3: 18 + 12 + 11 + 84 = 125 GPU 4: 19 + 13 + 20 + 92 = 144

^a^ End-to-end time: time that includes the initialization of the process and the execution of the reconstruction (×Speed-up: times faster than CPU reconstruction). ^b^ Recon time: time that includes only the execution of the reconstruction. ^c^ Number of NESTA iterations for reconstruction: (device: stage 1 + stage 2 + stage 3 + stage 4).

**Table 3:** Image quality metrics (SSIM and PSNR values) of the multi-device reconstructions for AF = 4 with the CPU reconstruction as reference.

SNR (dB)	SSIM			PSNR (dB)		
	2 GPUs	3 GPUs	4 GPUs	2 GPUs	3 GPUs	4 GPUs
*∞*	0.9654	0.9774	0.9766	41.55	42.46	43.23
24	0.9768	0.9781	0.9885	42.50	43.02	45.71
18	0.9832	0.9786	0.9909	40.81	40.06	43.75
6	0.9940	0.9902	0.9970	40.44	39.26	43.44

**Table 4:** Summary of VRAM memory usage and number of frames processed by different GPU configurations on server 1.

Memory Used				
VRAM	GPU ID	2 GPU ^a^	3 GPU ^a^	4 GPU ^a^
24,564 MiB	1	11,994 MB (180 MB)	8548 MB (180 MB)	6251 MB (180 MB)
24,564 MiB	2	11,994 MB (180 MB)	8994 MB (360 MB)	6697 MB (360 MB)
24,564 MiB	3	ND	7400 MB (180 MB)	6697 MB (360 MB)
24,564 MiB	4	ND	ND	6251 MB (180 MB)
**Number of Frames**				
	GPU ID	2 GPU ^b^	3 GPU ^b^	4 GPU ^b^
	1	44 (10 × 4 + 1 × 4)	32 (7 × 4 + 1 × 4)	24 (5 × 4 + 1 × 4)
	2	44 (10 × 4 + 1 × 4)	36 (7 × 4 + 2 × 4)	28 (5 × 4 + 2 × 4)
	3	ND	28 (6 × 4 + 1 × 4)	28 (5 × 4 + 2 × 4)
	4	ND	ND	24 (5 × 4 + 1 × 4)

^a^ Number of GPUs used: Total memory used (memory used for dependencies). ^b^ Number of GPUs used: Total number of frames on GPU (number of frames assigned to GPU + number of dependencies required on the GPU). Note: The GPUs used in this system have the same VRAM size. ND indicates that the setting does not apply.

**Table 5:** Summary of VRAM memory usage and number of frames processed in a 2-GPU configuration on server 2.

Memory Used		
VRAM	GPU ID	2 GPU ^a^
24,576 MiB	1	15,440 MB (180 MB)
16,384 MiB	2	8548 MB (180 MB)
**Number of Frames**		
	GPU ID	2 GPU ^b^
	1	56 (13 × 4 + 1 × 4)
	2	32 (7 × 4 + 1 × 4)

^a^ Number of GPUs used: Total memory used (memory used for dependencies). ^b^ Number of GPUs used: Total number of frames on GPU (number of frames assigned to GPU + number of dependencies required on the GPU).

**Table 6:** Summary of GPU utilization (median and maximum value) on server 1.

GPU Utilization (Median %)			
GPU ID	2 GPU ^a^	3 GPU ^a^	4 GPU ^a^
1	87%	86%	84%
2	86%	89%	82%
3	ND	77%	80%
4	ND	ND	79%
**GPU Utilization (Maximum %)**			
GPU ID	2 GPU ^b^	3 GPU ^b^	4 GPU ^b^
1	92%	90%	90%
2	92%	89%	85%
3	ND	86%	84%
4	ND	ND	87%

^a^ Number of GPUs used: Median value. ^b^ Number of GPUs used: Maximum value. Note: ND indicates that the setting does not apply.

## Data Availability

Data are contained within the article.

## Abbreviations

The following abbreviations are used in this manuscript:

AF	Acceleration Factor
bSSFP	Balanced Steady State Free Precession
cMRI	Dynamic Cardiac Magnetic Resonance Imaging
CPU	Central Processing Unit
CS	Compressed Sensing
CUDA	Compute Unified Device Architecture
FB	Free-Breathing
FOV	Field of View
GB	Gigabyte
GPU	Graphics Processing Unit
MRI	Magnetic Resonance Imaging
OpenCL	Open Computing Language
RAM	Random Access Memory
TB	Terabyte
TE	Echo Time of the MR Acquisition
TR	Repetition Time of the MR Acquisition
tTV	Temporal Total Variation
SSIM	Structural Similarity Index
VD-CASPR	Variable Density Cartesian Acquisition with Spiral Profile Order
VRAM	Video Random Access Memory

## Appendix A. Gradient of the Regularization Term

As commented in Section 3.2, the key to understanding data dependencies between the different devices is in the calculation of the term denoted as Λ within the gradient function $\nabla f(m^{k})$ (see Equation (3)), which must be evaluated at each iteration of the NESTA algorithm. This appendix details the evaluation of Λ for the global, non-partitioned, 5D reconstruction problem.

At the *k*-th iteration, $m^{k}$ denotes the set of image volumes, i.e., $m^{k}=\left\{m_{i,j}\right\}$, with pseudo-temporal indices $1\leq i\leq N_{r}$, and $1\leq j\leq N_{c}$, and with each $m_{i,j}$ defined over a 3D spatial domain: $X\subset R^{3}$. Then, we can evaluate $\Lambda =\Phi^{H}f_{\mu }^{\prime }\left(\Phi m^{k}\right)$ for a generic material point x∈X, with $m_{i,j}=m_{i,j}\left(x\right)$, and denote it as Λ(x). The term Λ involves operations along both the cardiac and respiratory dimensions. For the sake of simplicity, we will analyze each dimension separately by defining $\Lambda \left(x\right)=\Lambda^{c}\left(x\right)+\Lambda^{r}\left(x\right)$.

For the cardiac dimension, the application of the tTV operator ($\Phi_{c}$) can be expressed in matrix form as:

$$\begin{array}{c} m^{k}\left(x\right)\cdot \Phi_{c}=\left[\begin{array}{cccc} m_{1,1} & m_{1,2} & \cdots & m_{1,N_{c}}\\ m_{2,1} & m_{2,2} & \cdots & m_{2,N_{c}}\\ \vdots & \vdots & m_{i,j} & \vdots \\ m_{N_{r},1} & m_{N_{r},2} & \cdots & m_{N_{r},N_{c}} \end{array}\right]\underset{\Phi_{c}}{\underbrace{\left[\begin{array}{ccccc} -1 & 0 & 0 & \cdots & 0\\ 1 & -1 & 0 & \cdots & 0\\ 0 & 1 & -1 & \cdots & 0\\ 0 & 0 & 1 & \cdots & 0\\ \vdots & \vdots & \vdots & \ddots & \vdots \\ 0 & 0 & 0 & \cdots & 0\\ 0 & 0 & 0 & \cdots & -1\\ 0 & 0 & 0 & \cdots & 1 \end{array}\right]}}=\\ =\left[\begin{array}{cccc} m_{1,2}-m_{1,1} & m_{1,3}-m_{1,2} & \cdots & m_{1,N_{c}}-m_{1,N_{c}-1}\\ m_{2,2}-m_{1,1} & m_{2,3}-m_{2,2} & \cdots & m_{2,N_{c}}-m_{2,N_{c}-1}\\ \vdots & \vdots & \ddots & \vdots \\ m_{N_{r},2}-m_{N_{r},1} & m_{N_{r},3}-m_{N_{r},2} & \cdots & m_{N_{r},N_{c}}-m_{N_{r},N_{c}-1} \end{array}\right] \end{array}$$

Then, the derivative of the Huber function is element-wise evaluated, and the tTV adjoint operator ($\Phi_{c}^{H}$, defined as the conjugate transpose of $\Phi_{c}$) is applied as follows:

$$\begin{array}{c} \Lambda^{c}\left(x\right)=f_{\mu }^{\prime }\left(m^{k}\left(x\right)\cdot \Phi_{c}\right)\cdot \Phi_{c}^{H}=\\ =\left[\begin{array}{ccc} f_{\mu }^{\prime }\left(m_{1,2}-m_{1,1}\right) & \cdots & f_{\mu }^{\prime }\left(m_{1,N_{c}}-m_{1,N_{c}-1}\right)\\ f_{\mu }^{\prime }\left(m_{2,2}-m_{2,1}\right) & \cdots & f_{\mu }^{\prime }\left(m_{2,N_{c}}-m_{2,N_{c}-1}\right)\\ \vdots & \ddots & \vdots \\ f_{\mu }^{\prime }\left(m_{N_{r},2}-m_{N_{r},1}\right) & \cdots & f_{\mu }^{\prime }\left(m_{N_{r},N_{c}}-m_{N_{r},N_{c}-1}\right) \end{array}\right]\underset{\Phi_{c}^{H}}{\underbrace{\left[\begin{array}{cccccc} -1 & 1 & 0 & \cdots & 0 & 0\\ 0 & -1 & 1 & \cdots & 0 & 0\\ \vdots & \vdots & \vdots & \ddots & \vdots & \vdots \\ 0 & 0 & 0 & \cdots & -1 & 1 \end{array}\right]}}=\\ =\left[\begin{array}{cccc} -f_{\mu }^{\prime }\left(m_{1,2}-m_{1,1}\right) & f_{\mu }^{\prime }\left(m_{1,2}-m_{1,1}\right)-f_{\mu }^{\prime }\left(m_{1,3}-m_{1,2}\right) & \cdots & f_{\mu }^{\prime }\left(m_{1,N_{c}}-m_{1,N_{c}-1}\right)\\ -f_{\mu }^{\prime }\left(m_{2,2}-m_{2,1}\right) & f_{\mu }^{\prime }\left(m_{2,2}-m_{2,1}\right)-f_{\mu }^{\prime }\left(m_{2,3}-m_{2,2}\right) & \cdots & f_{\mu }^{\prime }\left(m_{2,N_{c}}-m_{2,N_{c}-1}\right)\\ \vdots & \vdots & \ddots & \vdots \\ -f_{\mu }^{\prime }\left(m_{N_{r},2}-m_{N_{r},1}\right) & f_{\mu }^{\prime }\left(m_{N_{r},2}-m_{N_{r},1}\right)-f_{\mu }^{\prime }\left(m_{N_{r},3}-m_{N_{r},2}\right) & \cdots & f_{\mu }^{\prime }\left(m_{N_{r},N_{c}}-m_{N_{r},N_{c}-1}\right) \end{array}\right] \end{array}$$

A similar analysis can be performed for operations along the respiratory dimension, leading to:

$$\Lambda^{r}\left(x\right)=\Phi_{r}^{H}\cdot f_{\mu }^{\prime }\left(\Phi_{r}\cdot m^{k}\left(x\right)\right)=\;\left[\begin{array}{cccc} -f_{\mu }^{\prime }\left(m_{2,1}-m_{1,1}\right) & -f_{\mu }^{\prime }\left(m_{2,2}-m_{1,2}\right) & \cdots & -f_{\mu }^{\prime }\left(m_{2,N_{c}}-m_{1,N_{c}}\right)\\ f_{\mu }^{\prime }\left(m_{2,1}-m_{1,1}\right)-f_{\mu }^{\prime }\left(m_{3,1}-m_{2,1}\right) & f_{\mu }^{\prime }\left(m_{2,2}-m_{1,2}\right)-f_{\mu }^{\prime }\left(m_{3,2}-m_{2,2}\right) & \cdots & \vdots \\ \vdots & \vdots & \ddots & \vdots \\ f_{\mu }^{\prime }\left(m_{N_{r},1}-m_{N_{r}-1,1}\right) & f_{\mu }^{\prime }\left(m_{N_{r},2}-m_{N_{r}-1,2}\right) & \cdots & f_{\mu }^{\prime }\left(m_{N_{r},N_{c}}-m_{N_{r}-1,N_{c}}\right) \end{array}\right]$$

where $\Phi_{r}$ and $\Phi_{r}^{H}$ are defined as:

$$\Phi_{r}=\left[\begin{array}{ccccccc} -1 & 1 & 0 & 0 & \cdots & 0 & 0\\ 0 & -1 & 1 & 0 & \cdots & 0 & 0\\ \vdots & \vdots & \vdots & \vdots & \ddots & \vdots & \vdots \\ 0 & 0 & 0 & 0 & \cdots & -1 & 1 \end{array}\right],\;\Phi_{r}^{H}=\left[\begin{array}{cccc} -1 & 0 & \cdots & 0\\ 1 & -1 & \cdots & 0\\ 0 & 1 & \cdots & 0\\ \vdots & \vdots & \ddots & \vdots \\ 0 & 0 & \cdots & -1\\ 0 & 0 & \cdots & 1 \end{array}\right].$$

Finally, we can conclude that

(A1) $$\begin{array}{c} \Lambda_{i,j}\left(x\right)=\Lambda_{i,j}^{c}\left(x\right)+\Lambda_{i,j}^{r}\left(x\right)=\\ =f_{\mu }^{\prime }\left(m_{i,j}-m_{i-1,j}\right)+f_{\mu }^{\prime }\left(m_{i,j}-m_{i,j-1}\right)-f_{\mu }^{\prime }\left(m_{i+1,j}-m_{i,j}\right)-f_{\mu }^{\prime }\left(m_{i,j+1}-m_{i,j}\right) \end{array}$$

with $f_{\mu }^{\prime }$, the gradient of the well-known Huber function, defined as

(A2) $$f_{\mu }^{\prime }\left(a\right)=\left\{\begin{array}{cc} a/\mu , & \mathrm{if}\;|a|\leq \mu \\ a/|a|, & \mathrm{otherwise}. \end{array}\right.$$

As commented in Section 3.2.3, the parameter μ changes for each stage (*s*) of the algorithm (see S.0 in Figure 5).

## References

[1] Menchón-Lara R.M., Simmross-Wattenberg F., Casaseca-de-la Higuera P., Martín-Fernández M., Alberola-López C. (2019). Reconstruction techniques for cardiac cine MRI. Insights Imaging.

[2] Turner R., Ordidge R., Haacke E., Liang Z.P. (2000). Technical challenges of functional magnetic resonance imaging. IEEE Eng. Med. Biol. Mag..

[3] Menchón-Lara R.M., del Val J.R., Godino-Moya A., Cordero-Grande L., Simmross-Wattenberg F., Martín-Fernández M., Alberola-López C., Cardoso M. (2017). An Efficient Multi-resolution Reconstruction Scheme with Motion Compensation for 5D Free-Breathing Whole-Heart MRI. Molecular Imaging, Reconstruction and Analysis of Moving Body Organs, and Stroke Imaging and Treatment.

[4] Feng L., Coppo S., Piccini D., Yerly J., Lim R., Masci P., Stuber M., Sodickson D., Otazo R. (2018). 5D whole-heart sparse MRI. Magn. Reson. Med..

[5] Yoon S., Nakamori S., Amyar A., Assana S., Cirillo J., Morales M.A., Chow K., Bi X., Pierce P., Goddu B. (2023). Accelerated Cardiac MRI Cine with Use of Resolution Enhancement Generative Adversarial Inline Neural Network. Radiology.

[6] Moya-Sáez E., Navarro-González R., Cepeda S., Pérez-Núñez Á., de Luis-García R., Aja-Fernández S., Alberola-López C. (2022). Synthetic MRI improves radiomics-based glioblastoma survival prediction. NMR Biomed..

[7] Becker S., Bobin J., Candès E.J. (2011). NESTA: A fast and accurate first-order method for sparse recovery. SIAM J. Imaging Sci..

[8] Khronos Group OpenCL (Version 3.0). https://www.khronos.org/opencl/.

[9] Simmross-Wattenberg F., Rodríguez-Cayetano M., Royuela-del Val J., Martin-Gonzalez E., Moya-Sáez E., Martín-Fernández M., Alberola-López C. (2018). OpenCLIPER: An OpenCL-based C++ Framework for overhead-reduced medical image processing and reconstruction on heterogeneous devices. IEEE J. Biomed. Health Inform..

[10] Wang H., Peng H., Chang Y., Liang D. (2018). A survey of GPU-based acceleration techniques in MRI reconstructions. Quant. Imaging Med. Surg..

[11] Smith D.S., Gore J.C., Yankeelov T.E., Welch E.B. (2012). Real-time compressive sensing MRI reconstruction using GPU computing and split Bregman methods. Int. J. Biomed. Imaging.

[12] Nam S., Akçakaya M., Basha T., Stehning C., Manning W.J., Tarokh V., Nezafat R. (2013). Compressed sensing reconstruction for whole-heart imaging with 3D radial trajectories: A graphics processing unit implementation. Magn. Reson. Med..

[13] Sabbagh M., Uecker M., Powell A.J., Leeser M., Moghari M.H. Cardiac MRI compressed sensing image reconstruction with a graphics processing unit. Proceedings of the 2016 10th International Symposium on Medical Information and Communication Technology (ISMICT).

[14] Shafique M., Qazi S.A., Omer H. (2023). Compressed SVD-based L+ S model to reconstruct undersampled dynamic MRI data using parallel architecture. Magn. Reson. Mater. Phys. Biol. Med..

[15] Lecoeur B., Barbone M., Gough J., Oelfke U., Luk W., Gaydadjiev G., Wetscherek A. (2023). Accelerating 4D image reconstruction for magnetic resonance-guided radiotherapy. Phys. Imaging Radiat. Oncol..

[16] Cuomo S., Michele P.D., Piccialli F. (2018). A (multi) GPU iterative reconstruction algorithm based on Hessian penalty term for sparse MRI. Int. J. Grid Util. Comput..

[17] Schaetz S., Voit D., Frahm J., Uecker M. (2017). Accelerated computing in magnetic resonance imaging: Real-time imaging using nonlinear inverse reconstruction. Comput. Math. Methods Med..

[18] Piccialli F., Cuomo S., De Michele P. (2013). A regularized MRI image reconstruction based on hessian penalty term on CPU/GPU systems. Procedia Comput. Sci..

[19] Schaetz S., Uecker M. (2012). A multi-GPU programming library for real-time applications. Proceedings of the Algorithms and Architectures for Parallel Processing: 12th International Conference, ICA3PP 2012, Proceedings, Part I 12.

[20] Murphy M., Alley M., Demmel J., Keutzer K., Vasanawala S., Lustig M. (2012). Fast l1-SPIRiT compressed sensing parallel imaging MRI: Scalable parallel implementation and clinically feasible runtime. IEEE Trans. Med. Imaging.

[21] Zhuo Y., Wu X.L., Haldar J.P., Hwu W.M.W., Liang Z.P., Sutton B.P. Multi-GPU implementation for iterative MR image reconstruction with field correction. Proceedings of the International Society for Magnetic Resonance in Medicine.

[22] Segars W.P., Sturgeon G., Mendonca S., Grimes J., Tsui B.M. (2010). 4D XCAT phantom for multimodality imaging research. Med. Phys..

[23] Prieto C., Doneva M., Usman M., Henningsson M., Greil G., Schaeffter T., Botnar R.M. (2015). Highly efficient respiratory motion compensated free-breathing coronary MRA using golden-step Cartesian acquisition. J. Magn. Reson. Imaging.

[24] NVIDIA Corporation NVIDIA RTX A5000 GPU Specifications. *Santa Clara, CA, EE. UU.*
**2024**. https://www.nvidia.com/en-us/design-visualization/rtx-a5000/.

[25] Advanced Micro Devices AMD EPYC 7513 CPU Specifications. *Santa Clara, CA, EE. UU.*
**2024**. https://www.amd.com/en/products/cpu/amd-epyc-7513.

[26] NVIDIA Corporation NVIDIA Quadro RTX 6000 GPU Specifications. *Santa Clara, CA, EE. UU.*
**2024**. https://www.nvidia.com/content/dam/en-zz/Solutions/design-visualization/quadro-product-literature/quadro-rtx-6000-us-nvidia-704093-r4-web.pdf.

[27] NVIDIA Corporation NVIDIA Quadro RTX 5000 GPU Specifications. *Santa Clara, CA, EE. UU.*
**2024**. https://www.nvidia.com/content/dam/en-zz/Solutions/design-visualization/quadro-product-literature/quadro-rtx-5000-data-sheet-us-nvidia-704120-r4-web.pdf.

[28] Intel Corporation Intel Xeon E5-2697 v4 CPU Specifications. *Santa Clara, CA, EE. UU.*
**2024**. https://www.intel.com/content/www/us/en/products/sku/91755/intel-xeon-processor-e52697-v4-45m-cache-2-30-ghz/specifications.html.

[29] Li A., Song S.L., Chen J., Li J., Liu X., Tallent N.R., Barker K.J. (2019). Evaluating modern gpu interconnect: Pcie, nvlink, nv-sli, nvswitch and gpudirect. IEEE Trans. Parallel Distrib. Syst..

[30] Butenhof D. (1997). Programming with POSIX Threads.

[31] Wang Z., Bovik A.C., Sheikh H.R., Simoncelli E.P. (2004). Image quality assessment: From error visibility to structural similarity. IEEE Trans. Image Process..

[32] Habermann T., Folk M. The hierarchical data format (HDF): A foundation for sustainable data and software. Proceedings of the AGU Fall Meeting Abstracts.

[33] (1993). Digital Imaging and Communications in Medicine (DICOM) Standard.

[34] Inati S.J., Naegele J.D., Zwart N.R., Roopchansingh V., Lizak M.J., Hansen D.C., Liu C.Y., Atkinson D., Kellman P., Kozerke S. (2017). ISMRM Raw data format: A proposed standard for MRI raw datasets. Magn. Reson. Med..

[35] Heye T., Knoerl R., Wehrle T., Mangold D., Cerminara A., Loser M., Plumeyer M., Degen M., Lüthy R., Brodbeck D. (2020). The energy consumption of radiology: Energy-and cost-saving opportunities for CT and MRI operation. Radiology.

